# TGFβ signalling pathway impacts brain metastases profiles in locally advanced colorectal cancer

**DOI:** 10.1007/s10585-024-10277-3

**Published:** 2024-03-18

**Authors:** Sven Jacob, Ilja Balonov, Vindi Jurinovic, Christian Heiliger, Tengis Tschaidse, Jörg Kumbrink, Thomas Kirchner, Jens Werner, Martin K. Angele, Marlies Michl, Jens Neumann

**Affiliations:** 1 Department of General, Visceral and Transplantation Surgery, University Hospital, LMU Munich, Munich, Germany; 2 Department of Medicine III, University Hospital, LMU Munich, Munich, Germany; 3 Institute of Pathology, Medical Faculty, Ludwig-Maximilians-University (LMU) Munich, Marchioninistr. 15, 81377 Munich, Germany; 4 The Institute for Medical Information Processing, Ludwig-Maximilians-University (LMU) Munich, Biometry, and Epidemiology, Munich, Germany

**Keywords:** Colorectal cancer, Hematogenous spread, Brain metastasis, Pathway analysis, Gene signature, TGFβ signalling pathway influences CRC brain metastasis

## Abstract

**Rationale:**

Colorectal Cancer (CRC) represents the third most common type of cancer in Germany and the second most common cancer-related cause of death worldwide. Distant metastases are still the main limit for patient survival. While liver metastases as well as peritoneal carcinomatosis can often either be resected or treated with systemic therapy, little options remain for brain metastases. Additionally, a number of studies has already investigated hepatic, peritoneal, pulmonary as well as continuing distant metastases in colorectal cancer. Yet, with respect to tumor biology and brain metastases, little is known so far.

**Material and methods:**

Two cohorts, M0 without distant spread and BRA with brain metastases were build. RNA was isolated from paraffin embedded specimen. Gene expression was performed by an RNA NanoString-Analysis using the nCounter® PanCancer Progression Panel by NanoString-Technologies (Hamburg, Germany). Results were analysed by principal component analysis, gene expression and pathway analysis using commonly available databases such as KEGG as benchmark for comparison.

**Results:**

We were able to determine a gene signature that provides a sophisticated group separation between M0 and BRA using principal component analysis. All genes with strong loading characteristics on principal component 1 were cross-referenced with the subsequently performed accurate gene set enrichment analysis (GSEA). The GSEA revealed a clear dysregulation of the TGFβ pathway in compared cohorts M0 and BRA. Interestingly, the targeted pathways analysis of the identified genes confirmed that in fact almost all strong loading genes of PC1 play a role in the TGFβ pathway.

**Conclusion:**

Our results suggest the TGFβ pathway as a crucial player in the development of brain metastases in primary CRC. In some types of colorectal cancer, downregulation of the TGFβ pathway might hinder primary colorectal cancer to metastasize to the nervous system. While the paradoxical functioning of the TGFβ pathway is still not fully understood, these shed light on yet another clinical implication of this complex pathway.

## Introduction

Colorectal Cancer (CRC) represents the third most common type of cancer in Germany and the second most common cancer-related cause of death worldwide. With more than 25% of CRC being diagnosed in distant metastatic state, one of the decisive factors for prognosis is the localization of distant metastases [1, 2]. Patients with liver metastases are often admissible for a curative approach. However, lacking possibilities of resection of metastases, which especially occurs in brain metastasis acts critically limiting for the prognosis [3]. Thus, it is crucial to understand the heterogenous organotropism of CRC and its molecular background leading to brain metastases.

Although there are standardized algorithms for liver and lung metastases, patients with brain metastases (BRA) are relatively rare and hence present a lack of standardized screening and treatment. With an average incidence of up to 4%, the rare but drastic state of BRA demands the need to be identified in early diagnostic processes since median overall survival with BRA is limited to 2–9 months [1, 4]. The complex interaction between genetic variability in patients with CRC and organotropism for liver, lung and peritoneal metastasis has been described in detail [5, 6]. Regarding brain metastases, this is not the case. Besides a mutation in KRAS, no clinically relevant gene mutations as well as no molecular pathways have been delineated to predict brain metastases in the CRC [7]. However, in patients with brain metastases from similarly common disease such as breast cancer and prostate cancer, several genes and pathways have been described to comprise somatic and germline mutations [8]. Among these, the TGFβ signalling pathway seems to play a crucial role. Respectively, there is an urgent need of further diversified genetic elucidation of patients with CRC and BRA as well as deeper investigation of genes and pathways involved. This is even more important because the most sensitive detection tool, the MRI, is rarely indicated and stays costly in terms of time, financial and technical resources for the health system in relation to this comparably rare condition [1].

In an attempt to identify frequently mutated genes in primary CRC with BRA, this study investigates specimens of locally advanced primary CRC without distant spread in comparison to primary CRC specimens leading to brain metastases. In respect to the heterogenous tumor biology of CRC, RNA NanoString analysis has been used to gain insights of differentially expressed gene. Principal component analysis, gene set enrichment analysis (GSEA) and pathway analysis from several widely used databases in both groups have been conducted. Furthermore, the results have been validated in corresponding data from the Kyoto Encyclopaedia of Genes and Genomes (KEGG).

## Material and methods

### Study population

Patients undergoing colorectal surgery at at the Department of General, Visceral and Transplantation Surgery at the Ludwig-Maximilian University Hospital Munich (Munich, Germany) were registered in a prospectively maintained database. Retrospectively, the patients were identified for the study population from this database using a predefined study protocol that was designed to address the research question at hand.

Criteria for study population were:

- Confirmed diagnosis of colorectal carcinoma by pathology
- Exclusion when additional malignant diagnosis other than colorectal carcinoma
- Exclusion of patients missing formalin-fixed paraffin-embedded (FFPE) tissue of the primary tumor
- Exclusion of patients presenting with Lynch-Syndrom and other hereditary diseases
- Exclusion of patients lost to follow up in the first 5 years

All CRC in the database were staged and documented by a pathologist according to the 7th edition AJCC TNM criteria from 2018. Follow up was achieved by cross-sectional imaging and continuous patient visiting. For investigational purposes, patients with hepatic and peritoneal metastases were not included in the present analysis, thus twelve patients, six from each group with and without brain metastases, were randomly selected for further characterization. Primary tumor RNA was isolated from FFPE specimens via microdissection as described previously. The study was carried out according to the recommendations of the local ethics committee of the Medical Faculty of the Ludwig-Maximilians-University Munich, Germany, which approved the study with protocol no. 19-966.

### Gene expressions analysis

This panel includes 770 genes particularly associated with the appearance and progression of distant metastases [9, 10]. Gen Set Enrichment Analysis (GSEA) was conducted by the JAVA program 2021. Gene set permutations were conducted 1000 times for each evaluation analogue to risk profiles previously described in numerous studies from the TCGA and ARG. The gene expression profiles and corresponding clinical information, such as age, gender, TNM classification and localization of distant metastases were maintained in the data base at hand. The primary GSEA included the whole genome analysis and was performed using the Broad Institute software. All values were compared to pathways from openly available databases: Reactome, Biocarta, Kyoto Encyclopedia of Genes and Genomes (KEGG), Gene Ontology (GO), Hallmark gene sets, oncogenic and immunologic signatures [11–13]. We adjusted all nominal p-values for multiple testing for each specific pathway data base with the Bonferoni-Holm method (q-value). P-values ≤ 0.05 and q-values ≤ 0.25 are assumed significant, p-values < 0.001 were considered highly significant.

### Statistical analysis

Statistical calculation was conducted using SPSS Version 25.0 (PASW, SPSS Inc. Chicago, IL, USA), MetaboAnalyst Version 5.0 (www.metaboanalyst.ca) and GraphPad Prism Version 9.1.2 (GraphPad Software, La Jolla, CA, USA). The data was presented as the mean for continuous variables and percentages for categorial variables. Associations and disparities were assessed by students t-test and a two-sided ${\chi }^{2}$ tests. Unsupervised principal component analysis (PCA) was applied to identify gene clusters. Functional groups of genes have been merged in synopsis with the statistical results and pathway analysis respectively. Clinicopathological data were delineated using one-way analysis of variance (ANOVA) and Tukey´s multiple comparisons test for multiple comparison correction. A two-sided p-value < 0.05 was considered statistically significant. Parameters with missing data (e.g., adjuvant chemotherapy) were included in the multivariate analysis by adding the category “unknown” to the respective variable to prevent listwise exclusion of cases from further analysis.

## Results

### Clinicopathological parameters

A total of 12 patients have been included in the complete dataset, six in each group. Median age of patients was 72.5 years (± 1.5 years) and the female to male ratio was seven female vs. five male patients. Regarding primary tumor location, there was no statistically significant difference between groups, the same applies to tumor grading. As defined per study protocol and confirmed by histology, all patients suffered from colorectal carcinoma. Likewise, every patient underwent systemic chemotherapy and resection. As expected, the difference in tumor stadium between M0 and BRA was statistically significant with all patients in BRA being classified as UICC 4 (Tables 1 and 2).

**Table 1:** Clinicopathological parameters

Variable	M0 n (%)	BRA n (%)	p-value
Patients	6	6	
Medium age	71	74	0.72
Sex			
Female	4 (66.6)	3 (50.0)	0.75
Male	2 (33.4)	3 (50.0)	
Location of PT			
Right colon	2(33.3)	4 (66.7)	0.40
Left colon	4 (66.7)	2 (33.3)	
UICC			< 0.05
I	0 (0.0)	0 (0.0)	
II	0 (0.0)	0 (0.0)	
III	6 (100.0)	0 (0.0)	
IV	0 (0.0)	(100.0)	
Grading			
Low grade	3 (50.0)	3 (50.0)	0.99
High grade	3 (56.9)	3 (50.0)	
Chemotherapy			
Yes	6 (100.0)	6 (100.0)	> 0.9
No	0 (0.0)		0 (0.0)

Bra = brain metastases, M0 = no metastases

**Table 2:** Mutational status

Sample	Group	KRAS	NRAS	BRAF	MSI	T	N	M	G
1	BRA	mut (Ex2)	wt	wt	MSS	3	1	1	3
2	BRA	mut (Ex2)	wt	wt	MSS	4	1	1	2
3	BRA	mut (Ex2)	wt	wt	MSS	4	2	1	3
4	BRA	wt	wt	mut	missing	3	2	1	2
5	BRA	wt	wt	wt	MSS	4	2	1	2
6	BRA	wt	wt	wt	MSS	3	2	1	2
7	M0	missing	missing	missing	missing	3	1	0	3
8	M0	wt	missing	wt	missing	3	2	0	3
9	M0	missing	wt	missing	MSS	3	1	0	3
10	M0	missing	missing	missing	missing	3	1	0	2
11	M0	wt	mut	missing	missing	3	2	0	2
12	M0	mut (Ex2)	fehlt	missing	missing	3	1	0	2

## Gene expression

### Overall principal component analysis

**Fig. 1:**
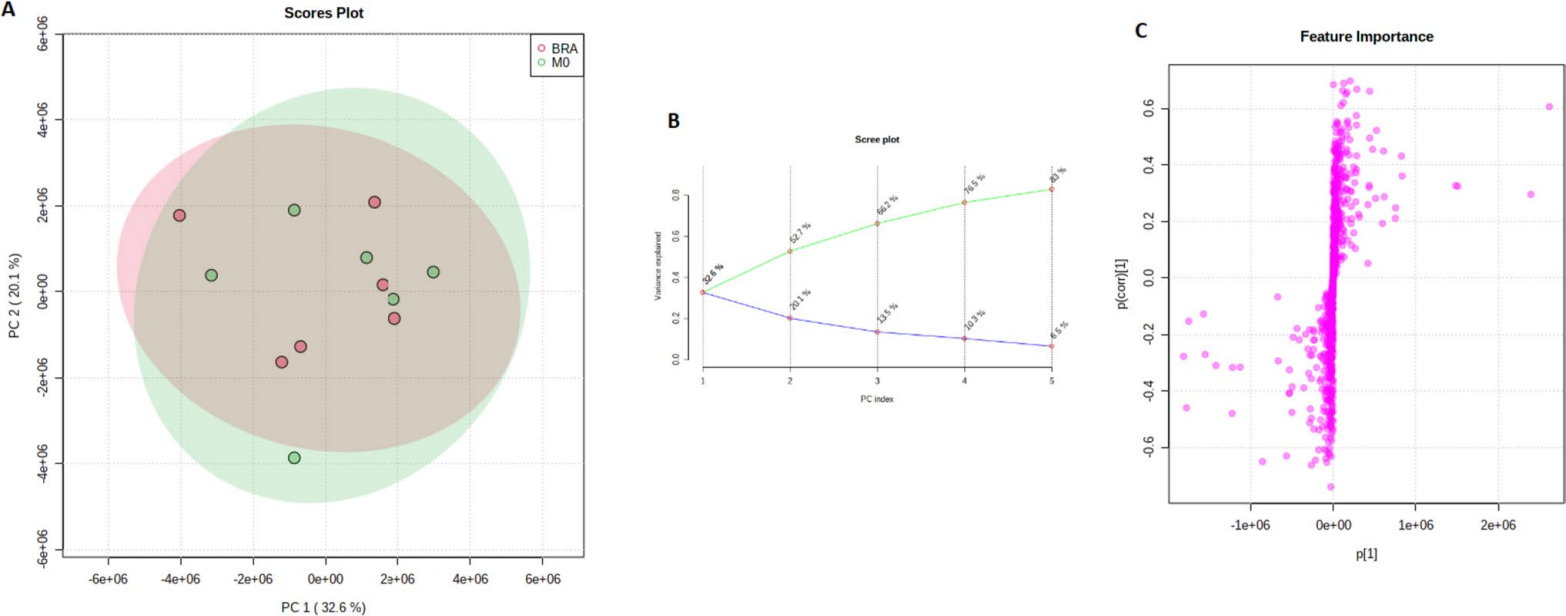
**A** showing PCA Scores plot between the PCs is pairwise providing an overview of the various separation patterns among the significant PCs. The explained variances are shown in brackets. **B** is the Scree plot showing the variances explained by the calculated PCs. Scree plot shows the variance explained by PCs. The green line on top shows the accumulated variance explained; the blue line underneath shows the variance explained by individual PC. **C** delineates S-plot showing the variable importance in a model, combining the covariance and the correlation (p(corr)) loading prole. (Color figure online)

At first, a PCA with 770 genes has been examined in order to identify principal components potentially indicating genes, which separate the cohorts BRA and M0. Kaiser–Meyer–Olkin Measure of Sampling Adequacy (KMO = 0.678 and Chi-quadrat (120) = 420, p < 0.02) displays validity of the calculated principal component analysis. Principal component 1 explains 32.6% of variance. PC2 explains 20.1% of variance in given calculation. Further components tested did not add any additional value. The corresponding Scree plot in Fig. 1B shows five PCs with an Eigenvalue > 1. Each point in the plot delineates a tissue sample. However, principal component analysis did not show a relevant genetic separation. With no distinct elbow spot in the plot, further evaluation of given model does not indicate if a two-, three-, four- or five-factor analysis will facilitate an adequate result. In an S-plot, a group of genes have been identified to separate from the main group graphically. Reckoning of genes with notably low or high covariance, a further examination on significance will follow the previous analysis.

### Analysis of differentially expressed gens: BRA vs. M0

**Fig. 2:**
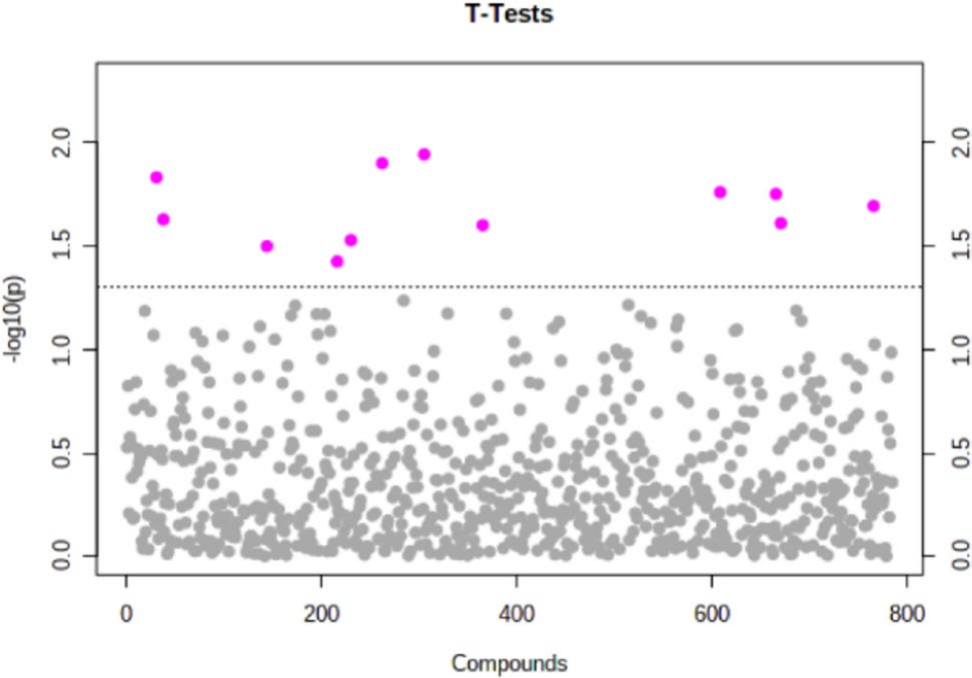
Shows identified five features correlating positively and seven features correlating negatively with the corresponding PCs

**Fig. 3:**
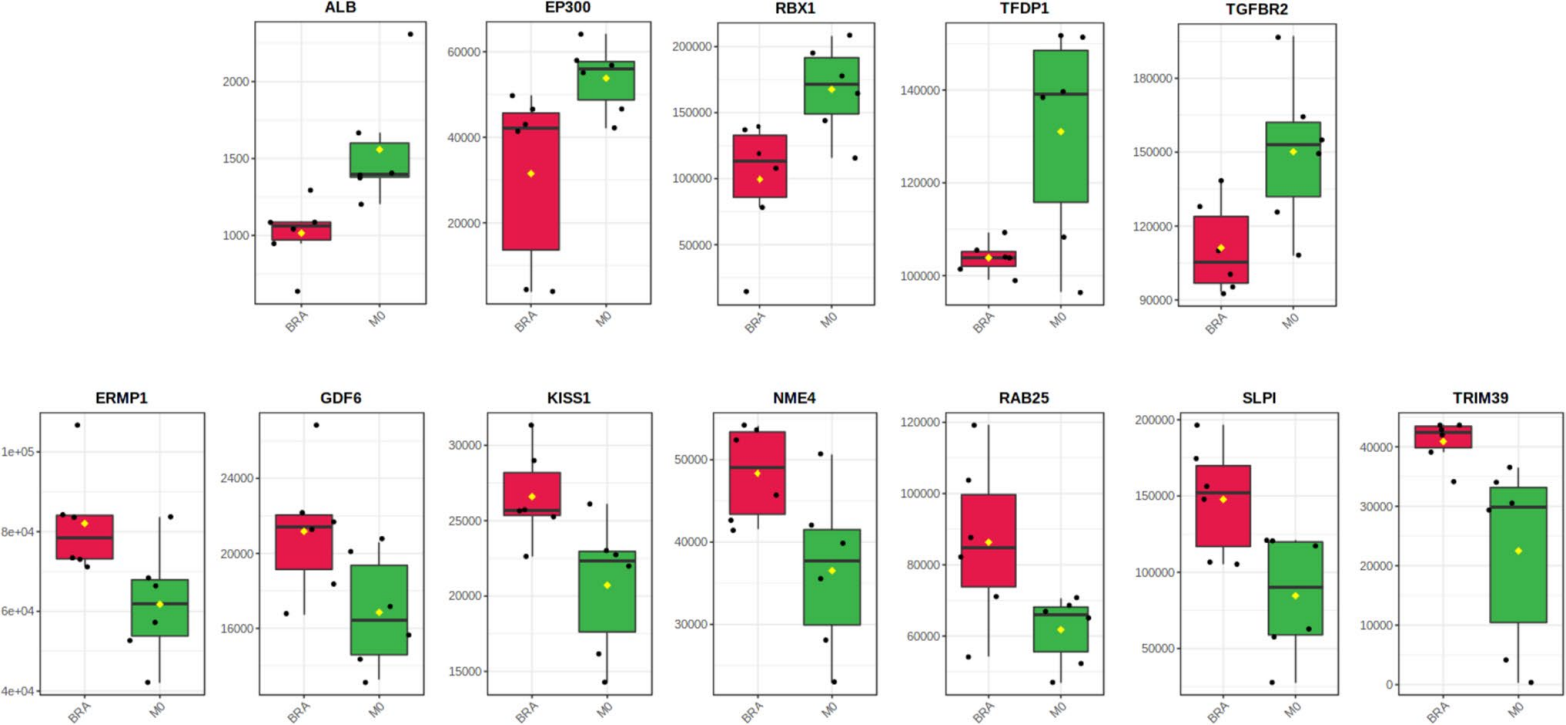
Shows identified five features correlating positively and seven features correlating negatively with the corresponding PCs

Based on previous results from multivariate statistics, the Student’s T-test was applied to elucidate for potential genes that were significantly different between the BRA and M0 tissue samples (p < 0.05, Benjamini–Hochberg false discovery rate). Here 12 genes have been identified with a significant difference between the two groups based on their individual relative expression. The characterization of these genes is described in Fig. 2 and Table 3. Further analysis continued with only these detected genes. Within the twelve significantly different genes, five genes correlate negatively with BRA while seven correlate positive with BRA as shon in Fig. 3.

**Table 3:**
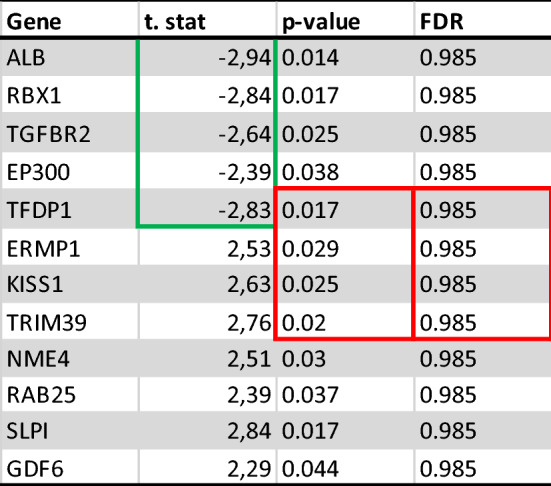
**A** shows the important features identified by t-tests

Important features selected by t-tests with threshold 0.05. Purple circles represent features above the threshold. Note the p values are transformed by -log10 so that the more significant features (with smaller p values) will be plotted higher on the graph. **B** includes statistical characteristics of the 12 identified genes

### Correlation of differentially expressed genes

**Fig. 4:**
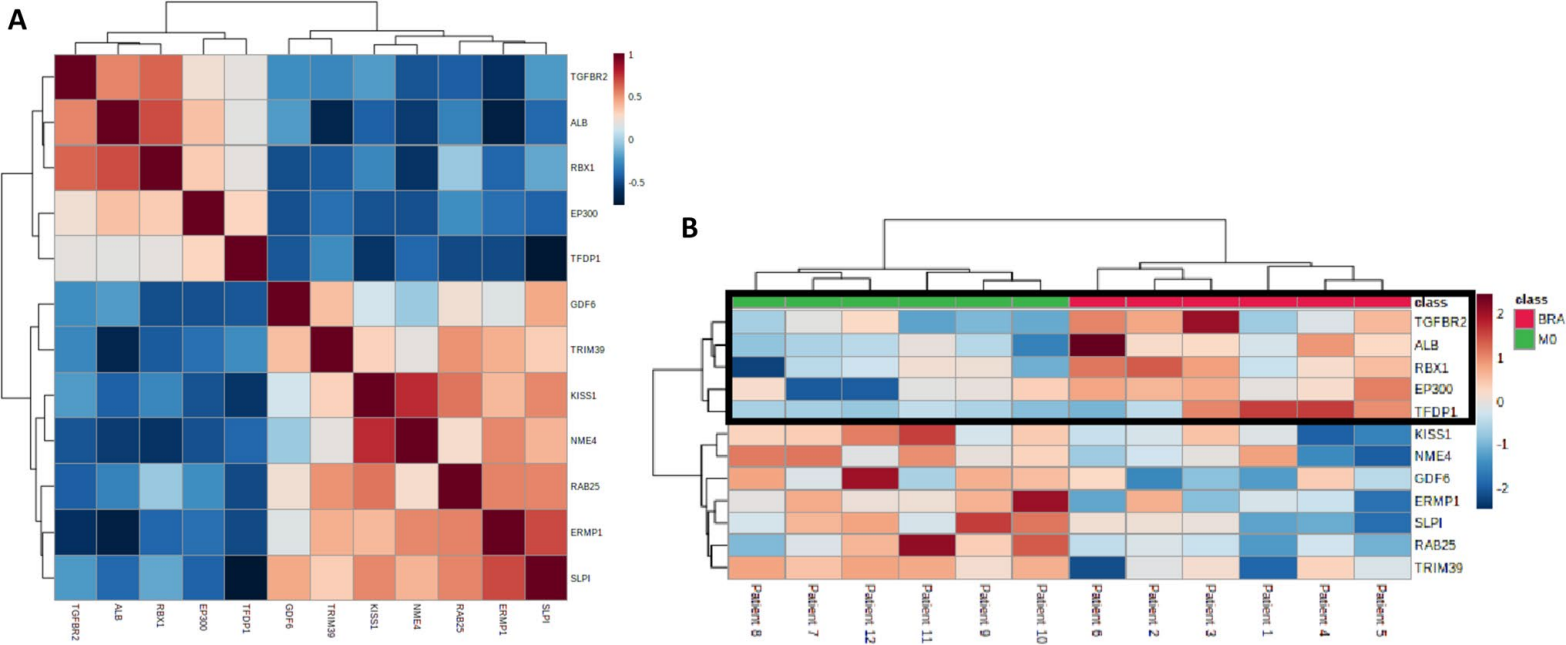
**A** shows the clustering result in the form of a heatmap. **B** overall correlation heatmap between the significant genes and the cohort without prior clustering

To elucidate a potential correlation between significantly different expressed genes and the cohorts at hand, a Heatmap containing the expression of the respective genes has been performed. Horizontal and vertical columns display relative expression of each gene delineating the correlation between the genes. Each bar in the columns represents the expression intensity. For example, the blue scale indicates a decreased level, while the red scale indicates an increased level. The dendrogram on the left was codirected based on the genetic intensity expression profiles (Fig. 4A). Moreover, in the next step (Fig. 4B) an investigation of potential correlation between identified genes and both patient groups were performed. Here the gene expression profile accurately reflects the discrimination of the cohort M0 and BRA without prior codirection. The five previously identified genes, which are increased in BRA group and load on TGFβ pathway have been shown to correlate within each other. Also, a correlating antecedence of TGFBR2, ALB and EP300 for the BRA group appears to reveal as well.

### Principal component analysis focusing on differential gene expression profiles

**Fig. 5:**
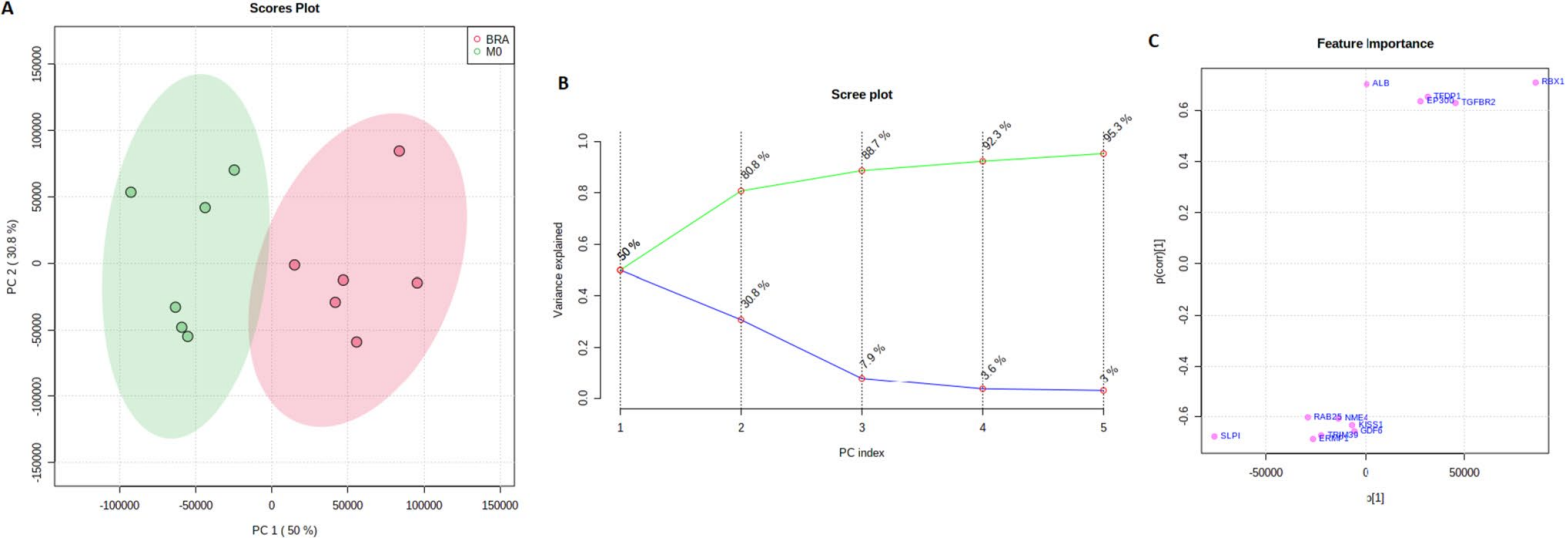
**A** visualizes PCA Scores plot between the PCs is pairwise providing an overview of the various separation patterns among the significant PCs. The explained variances are shown in brackets. **B** depicts the Scree plot showing the variances explained by the calculated PCs. Scree plot shows the variance explained by PCs. The green line on top shows the accumulated variance explained; the blue line underneath shows the variance explained by individual PC. **C** displays PCA loadings S-plot showing the variable importance in a model, combining the covariance and the correlation (p(corr)). (Color figure online)

As a PCA with 770 genes failed to separate the BRA group from the M0 group, the same method was applied with the significantly different genes as shown in Fig. 5. The second PCA with twelve genes distinguished the cohort into two separate groups. Kaiser–Meyer–Olkin Measure of Sampling Adequacy (KMO = 0.841 and Chi-quadrat (120) = 721, p < 0.02) betokens validity of the calculated principal component analysis. Principal component 1 explains 50% of variance. PC2 explains 30.8% of variance in given calculation. Thus, a more than satisfying distinction between both cohorts has been achieved. Corresponding Scree plot shows two PCs with an Eigenvalue > 1. A distinct elbow spot after PC2 indicates certainty of further analysis as a two-factor analysis. In a S-plot, the predefined genes have been confirmed to be characterized with high correlation and covariance with the separation of the cohort into the clinically different groups. Interestingly, the characterization of the twelve significantly different genes on their corresponding principal component delineates an inhomogeneous allocation of loading on each component.

Despite the correlation of above mentioned differentially expressed genes, functional gene groups have been characterized by specific loadings of each gen on the principal components using the Cohen loading as a cut off, where larger than 0.5 is assumed as strong loading [14]. In respect to the identified genes, ALB, TGFBR2, RBX1, TFDP1 and EP300 exhibited critically positive or negative loading on principal component 1. In comparison, three genes have shown clear positive loading on principal component 2. Nonetheless, with given results from PC1 explaining more that 50% of the variance between both groups, more detailed investigation of PC1 and its functional group of genes by pathway analysis was indicated. Loading are summarized in Table 4.

**Table 4:**
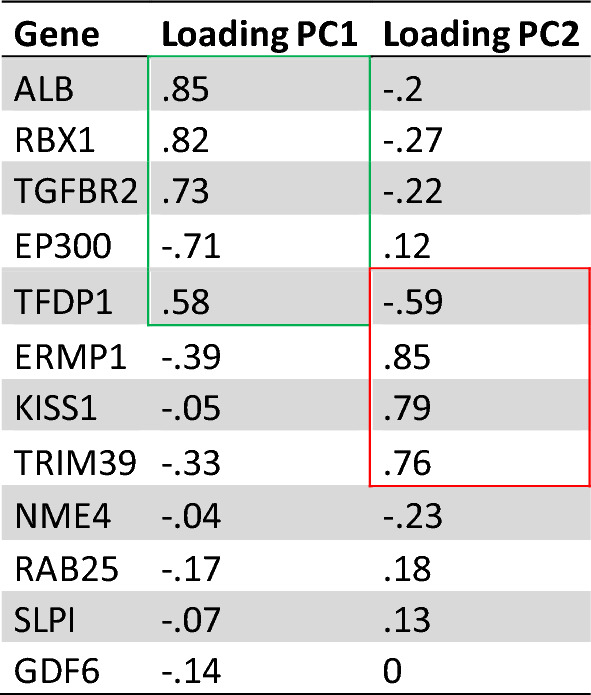
Shows the loading of identified significant features on PC1 and PC2

Green and red marks strong (> .5) in cohan scale

## GSEA analysis

### Tested databases and pathways

The set of genes identified with the NanoString analysis were further evaluated by GSEA. This pathway analysis, as usual, considered the complete set of genes without regard to single gene expression values. Furthermore, GSEA and NanoString results were compared to several commonly available databases namely Biocarta, Gene Ontology (GO), Reactome, Kyoto Encyclopedia of Genes and Genomes (KEGG), Hallmark gene sets, oncogenic and immunologic signatures. Each database, the total number of potential pathways of each database as well as the number of tested pathways are described in Table 4.

**Fig. 6:**
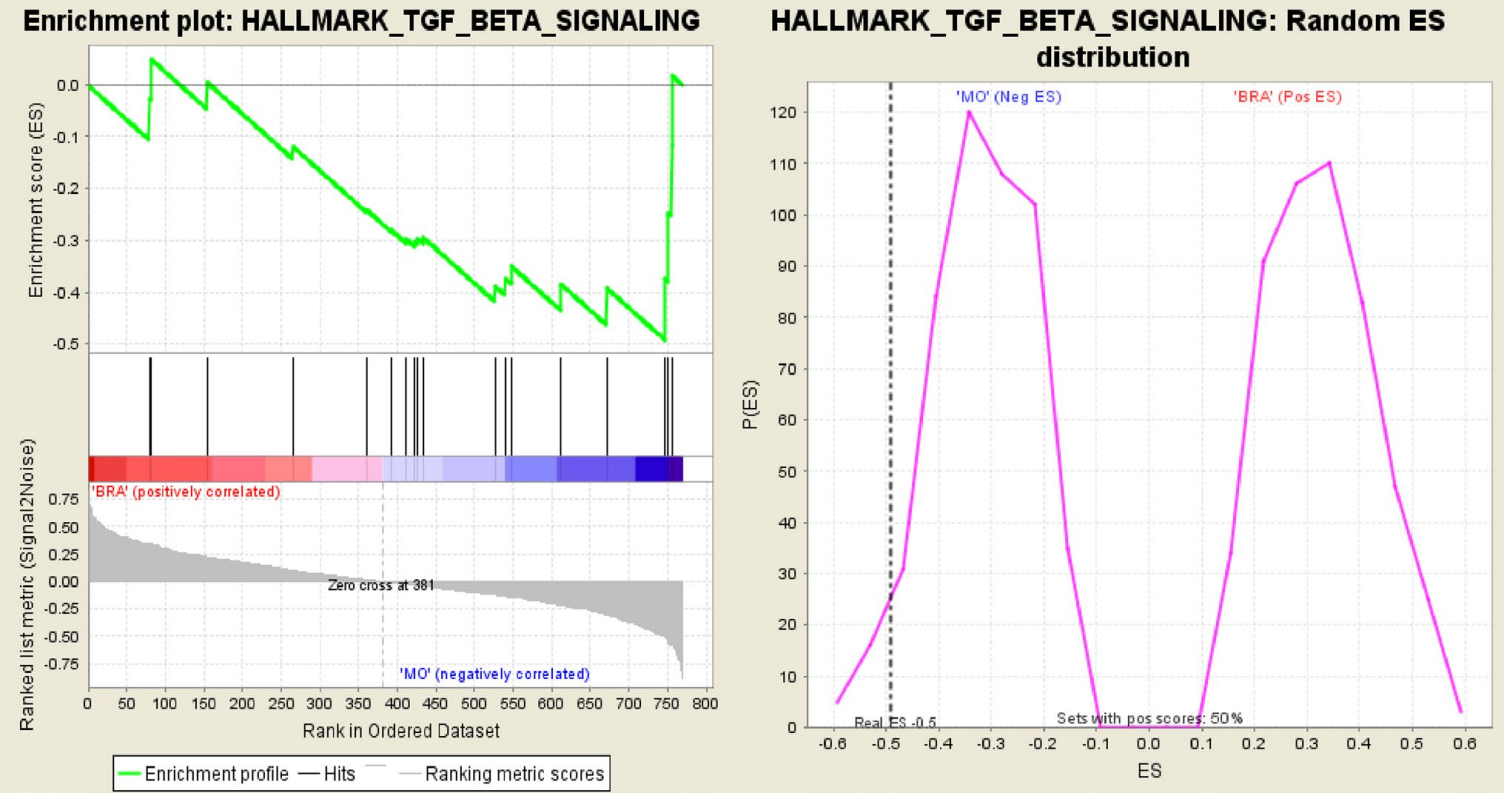
Enrichment plot for the TGFβ signalling pathway from Hallmark database. Here the enrichment curve indicates a significant alteration in comparison to the M0 group

**Fig. 7:**
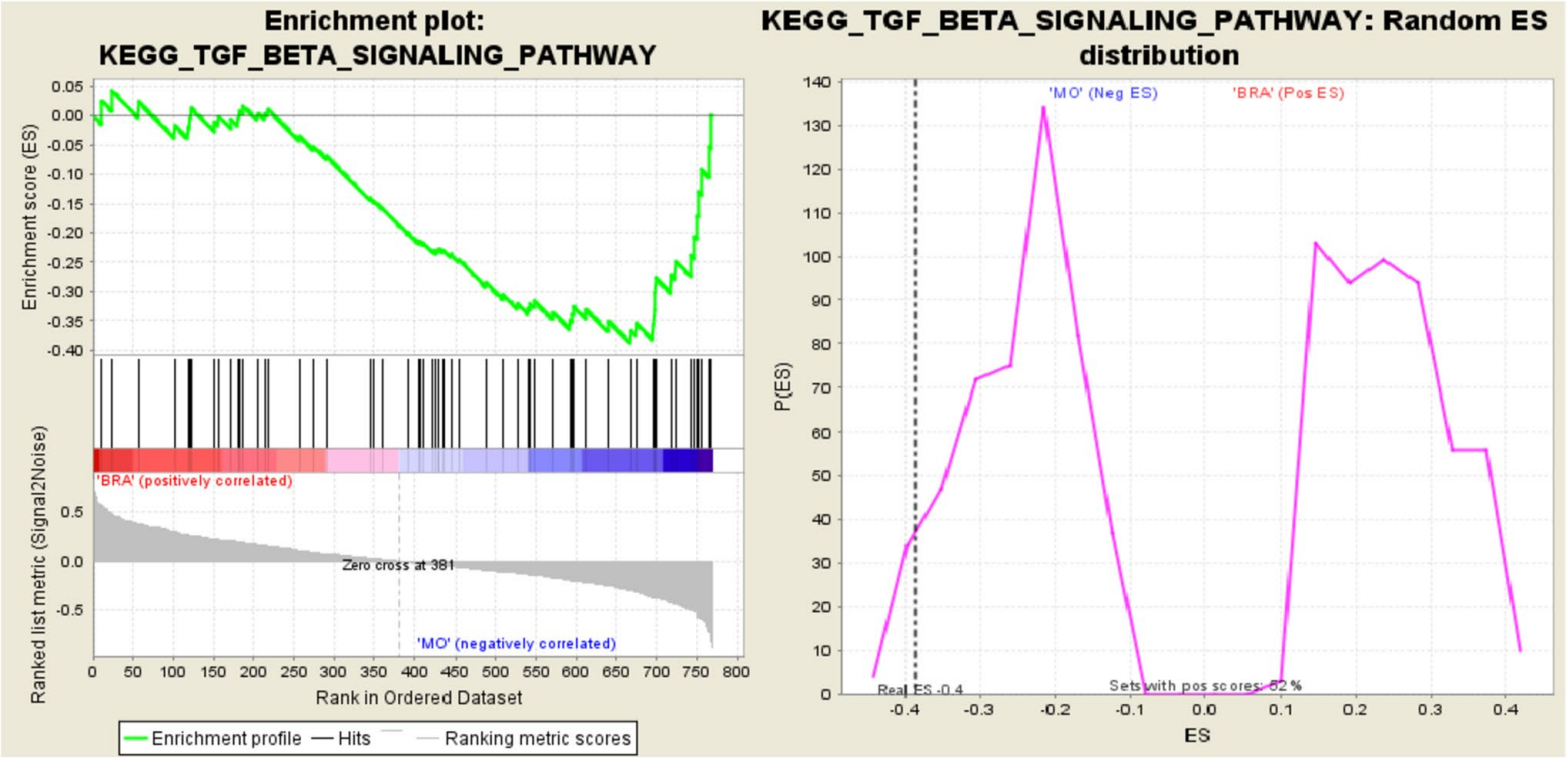
Enrichment plot for the TGFβ signalling pathway from Hallmark database. Here the enrichment curve indicates a significant alteration in comparison to the M0 group

GSEA was applied to compare M0 and BRA group to display potential profiles of both groups and herewith delineate risk profiles. Several pathways proved to be significantly different expressed between both groups BRA and M0 (Table 5). To further elucidate on important genes influencing the process of brain metastases, we cross-referenced the high-risk gene set of this analysis with all significantly different expressed pathways (Table 6). Of those, all genes critically loading on PC1 also play a role in the TGFβ-pathway. The TGFβ-pathway itself proved to be significantly different expressed using not only the Hallmarks (Fig. 6) but also the KEGG (Fig. 7) pathway database (normalized enrichment score (NES) = −1.5 p < 0.04, q = 0.20; NES = −1.7, p < 0.04, q = 0.16 respectively). Of those 5 genes loading on PC1, four genes, namely TGFßR2, TFDPI, RBX1, and EP300 even contributed to the core enrichment score of the TGFβ-pathway indicating a major influence on its functioning. Thus, in tumours leading to brain metastases (BRA) compared to M0, the TGFβ signalling is significantly dysregulated.

**Table 5:** Tested databases and pathways

Database	Potential pathways (n)	Tested pathways (n)	n	p-value	q-value
Reactome pathways	674	58			
INNATE IMMUNE SYSTEM			26	0.014	0.22
KEGG pathways	186	57			
CHEMOKINE SIGNALING			45	0.014	0.21
NOD LIKE ECEPTOR SIGNALING			16	0.024	0.17
TGF BETA SIGNALING			60	0.043	0.17
LEISHMANIA INFECTION			21	0.025	0.19
WNT SIGNALING			35	0.058	0.21
COLORECTAL CANCER			30	0.057	0.23
ADHERENS JUNCTION			25	0.11	0.21
Hallmarks pathways	50	24			
INTERFERON GAMMA_RESPONSE			26	< 0.001	0.16
ALLOGRAFT REJECTION			41	0.002	0.094
TGF BETA SIGNALING			19	0.046	0.21
Biocarta	217	14			
ALK			16	0.039	0.21
MAPK			19	0.024	0.14
PPARA			18	0.045	0.20
CTCF			15	0.09	0.23
BIOPEPTIDES			16	0.12	0.24
Immunologic signatures	4872	1177			
GSE29618			15	< 0.001	0.21
GSE9988			20	< 0.001	0.22
GSE43863			16	0.01	0.17
GSE8685			16	0.004	0.19
GSE12366			19	0.004	0.18

**Table 6:**
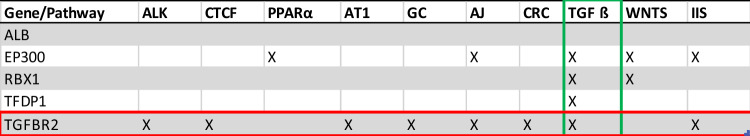
significant genes and significant pathways

Genes involved in corresponding pathways marked with X

## Discussion

Recently, research of distant metastases in CRC patients increased exponentially. A number of studies has already investigated hepatic, peritoneal, pulmonary as well as continuing distant metastases in colorectal cancer [5, 15]. Yet, with respect to tumor biology and brain metastases, little is known so far.

The aim of this work was to analyse genetic differences and commonalities between patients without distant metastases and patients with brain metastases from colorectal adenocarcinoma. Therefore, RNA NanoString-Analysis was applied. We elucidated on comparative differences in the genetical profiles of M0 and BRA tumour tissues by using an unsupervised principal component analysis (PCA). The final PCA was performed using the complete set of significantly different expressed genes between M0 and BRA. We were able to determine a gene signature that provides a sophisticated group separation between M0 and BRA (Fig. 4). The consecutive scree- and S-plots in Fig. 4 confirm this analysis. Moreover, the scree-plot suggested that only PC1 was able to explain more that 50% of the variance between BRA and M0. The unsupervised heat map in Fig. 5B illustrates a clear cluster pattern dividing M0 from BRA. All genes with strong loading characteristics on PC1 were therefor cross-referenced with the subsequently performed accurate gene set enrichment analysis (GSEA). The GSEA revealed a clear dysregulation of the TGFβ pathway in compared cohorts M0 and BRA (Figs. 6 and 7). Interestingly, the targeted pathways analysis of the identified genes confirmed that in fact almost all strong loading genes of PC1, namely ALB, RBX1, TGFßR2, EP300 and TFDP1, play a role in the TGFβ pathway suggesting the TGFβ pathway as a crucial player in the development of brain metastases in primary CRC.

The TGFβ pathway signalling pathway is said to have a paradox influence on tumour progression and metastases, a fact well known as the TGFβ paradox. Recent literature argues that physiological TGFβ upregulation triggers induction of apoptosis and proliferation as well as cell cycle arrest in early stage cancer cells [16, 17]. In healthy tissue likewise, TGFβ inhibits epidermal growth and cell transition and thus shows an anti-tumour effects. These results are supported by Bakir et al. 2020, who argue that a TGFβ-R2 deficiency, a core TGFβ pathway regulator, leads to increased inflammatory burden and tumor progression via higher levels of tumor necrosis factor-α (TNF-α), interleukin (IL)-8 and interferon (IFN)-γ [17]. These findings might directly explain the observed overregulation of the TGFβ pathway in our M0 cohort. This might underline that under certain circumstances, the TGFβ pathway might inhibit metastatic potential. Further, the TGFBR2 gene is downregulated in BRA in our gene expression analysis. This result is supported by its negative loading on PC1 in the principal component analysis of this stud. Li et al. 2017 highlighted a direct link to our findings by real-time PCR results in non-small cell lung cancer tissue. They could indeed correlate a repression of TGFBR2 with more distant metastases and tumor growth [18].

TGFβ inactivation in malignant colon cells has also proved to boost malignant potential via the MAPK and Wnt-ß-catenin pathway [19]. Even after curative resection, a disruption in TGF-β signaling results in a much more progressive phenotype thus limiting patient prognosis [20]. Notably, these results have been shown to be true especially for advanced cancer stages. The fact that in our cohort, overrepresentation of the TGFβ signalling might act preventive regarding brain metastases might serve as new insights as to how TGFβ signalling influences tumor behaviour.

Contrary to these findings, others argue that even in low-stage cancer cells, TGFβ can induce tumour progression. While this assumption does not contradict our findings, it supports the above mentioned TGFβ paradox. Additionally, this paradox states that in later stage cancer types, the opposite such as genomic instability and immune evasion as well as tumorigenic alterations in peritumoral stroma cells is possible. In that context, the TGF-β pathway is associated with changes in Erk, MAPK and SMAD signaling [16]. Contradictory to that, however, are the finding of Bacman et al. 2007. They state that a loss of key regulators of the TGFβ pathway, TGFß-R1 and R2, results in increased lymph node metastases and shorter survival rates [21]. They further mention that peritumoral stromal TGF-beta R2 even serve as an independent prognostic marker for survival. These results are supported by Hussain et al. 2018. Their hypothesis was that higher levels of TGFβ dependent IL-23 lead to less macrophage associated metastases in pancreatic cancer [22]. They therefore injected NGS mice with IL-23, macrophages and TGFβ and could observe less metastatic potential and higher levels of IL-23, macrophages and TGFβ in the long-term survivor group [22]. Even stronger in line with our finding that at least parts of the TGFβ pathway prevent brain metastases are the results of Okita et al. 2018. While they confirm that TGFβ-signalling mutations might enhance EMT and metastases in some CRC patients, they point out other subtypes in which TGF-βRII actively hinders EMT and metastases thus leading to a better prognosis [2]. In seems very interesting to further elucidate on those subtypes regarding brain metastases and TGFβ pathway expressions.

The context dependent, by times paradox biological implications within the TGFβ pathway ask for further investigation. It is still unclear why this pathway can enhance and hinder tumor progression in the same type of cancer. While we could point out that the TGFβ signalling might reduce brain metastases, above mentioned research can also indicate that it can just as well lead to more metastatic potential. A question which again can be summed up as the paradox functioning of the TGFβ pathway. Taken together the demonstrated results and pre-existing evidence, specific mutations as well as up or downregulations in the TGFβ pathway have been re-assessed to influence the development of distant metastases in the central nervous system. Undoubtedly, further elucidation on why and how the process of brain metastases is influenced by the TGFβ pathway is required. More so because it remains unclear which regulating genes play a core role in the biologically complex network facilitating brain metastases. Patient survival can shrink down to 2–10 months after the development of brain metastases. Further, the gold standard for brain metastases detection is still the MRI, notably not a routine diagnostic, complex and costly. We therefore think that our research can be an efficient starting point to elucidate on gene signatures and biomarkers for early detection of brain metastases.
